# Simple Green Route to Performance Improvement of Fully Bio-Based Linseed Oil Coating Using Nanofibrillated Cellulose

**DOI:** 10.3390/polym9090425

**Published:** 2017-09-07

**Authors:** Stefan Veigel, Eva-Marieke Lems, Gerhard Grüll, Christian Hansmann, Thomas Rosenau, Tanja Zimmermann, Wolfgang Gindl-Altmutter

**Affiliations:** 1 Department of Material Sciences and Process Engineering, BOKU—University of Natural Resources and Life Sciences, Vienna, Konrad Lorenz Straße 24, 3430 Tulln, Austria; eva.lems@boku.ac.at (E.-M.L.); c.hansmann@kplus-wood.at (C.H.); wolfgang.gindl-altmutter@boku.ac.at (W.G.-A.); 2 Holzforschung Austria, Franz Grill Straße 7, 1030 Vienna, Austria; g.gruell@holzforschung.at; 3 Kompetenzzentrum Holz GmbH, Altenberger Straße 69, 4040 Linz, Austria; 4 Department of Chemistry, BOKU—University of Natural Resources and Life Sciences, Vienna, Konrad Lorenz Straße 24, 3430 Tulln, Austria; thomas.rosenau@boku.ac.at; 5 Applied Wood Materials Laboratory, EMPA—Swiss Federal Laboratories for Materials Science and Technology, Überlandstraße 129, 8600 Dübendorf, Switzerland; tanja.zimmermann@empa.ch

**Keywords:** wood coating, linseed oil, nanocellulose, surface modification, wear resistance

## Abstract

Due to their bio-based character, oil-based coatings become more and more prevalent in wood surface finishing. These coatings impart appealing optical and haptic properties to the wood surface, but lack sufficient protection against water and mechanical influences. The present study reports a simple green route to improve the performance of linseed oil coating by the addition of nanofibrillated cellulose (NFC). In order to achieve surface chemical compatibility with linseed oil, NFC was chemically modified with acetic anhydride and (2-dodecen-1-yl)succinic anhydride, respectively, using propylene carbonate as a solvent. NFC/linseed oil formulations were prepared and applied to wood substrates. The wear resistance of oil-coated wood surfaces was assessed by a newly developed test combining abrasive loading with subsequent contact angle measurement. As revealed by infrared and nuclear magnetic resonance (NMR) spectroscopy, as well as X-ray diffraction (XRD), NFC has been successfully modified without significantly affecting the structure of cellulose. In abrasion tests, all NFC-modified oil coatings performed better than the original oil. Interestingly, NFC only suspended in propylene carbonate, i.e., without chemical modification, had the strongest improvement effect on the coating’s wear resistance. This was primarily attributed to the loose network structure of this NFC variant which effectively prevents the oil from penetration into the wood surface, thus forming a protective NFC/oil composite layer on the wood surface.

## 1. Introduction

By volume, the forest products industry is the most significant provider of raw materials for sustainably-produced chemicals and materials. In order to preserve the natural, bio-based character of the raw material wood throughout the entire production process up to the final product, bio-based adhesives [1,2] and coatings are of high interest both to manufacturers and consumers of wood-based products. While solvent-based systems have been dominant in film-forming wood coatings over a long period of time, environmental concerns have led to a strong increase in the use of waterborne coatings [3,4]. As an alternative to film-forming coating systems, oil-based coatings also offer a certain protection to wood surfaces. The use of linseed oil in wood coatings, which can be tracked back many centuries [5,6], has attracted renewed interest in high-quality solid wood furniture and wood flooring due to the unique open-porous character and favourable haptics of oil-treated wood. Linseed oil penetrates into the wood surface and dries by an autoxidation process [6]. While linseed oil provides highly-appreciated optics and haptics to wood surfaces, protection against water through surface hydrophobisation and protection against mechanical wear are provided only to a minimum. An improvement of the wear resistance of oil-based wood coatings is therefore of high interest. If full preservation of the bio-based character of linseed oil coating is a prerequisite, and chemical modification of the oil for improved properties is, therefore, not an option, addition of inorganic nanoparticles, e.g., ZnO, TiO_2_, nanoalumina, or nanosilica may be a potential route for performance enhancement [7,8]. For instance, Nejad et al. [9] showed that the addition of 1% inorganic nanoparticles significantly increased the abrasion resistance of a vegetable oil-based coating. Furthermore, nanoclay was found to reduce the drying time of coating by 37% but, at the same time, it had a negative effect on coating viscosity, which was increased by five times. In the context of wood products, cellulose-based nanostructures are particularly interesting, since they stem from the same raw-material. Two main classes of cellulose-based nanostructures may be discerned [10]. First, mechanical fibrillation by means of high shear forces yields nanofibrillated cellulose (NFC). NFC is characterised by a network-like, entangled structure, with characteristic fibril diameters between 10 and 50 nm and a length of >1 µm (high aspect ratio). Second, acid hydrolysis of non-crystalline domains in cellulose yields cellulose nanocrystals (CNC). CNC is of smaller characteristic diameter (5–10 nm) and significantly shorter (100–200 nm) than NFC (lower aspect ratio). Cellulose nanoparticles show high tensile strength and stiffness [11]. The distinctly polar surface of cellulose complicates processing of nanocellulose with non-polar solvents and polymers [12], necessitating chemical surface modification [13] or other measures to improve compatibility. The high specific surface area of nanocellulose greatly increases the viscosity of liquids even at small concentrations of a few percent [14,15,16]. Due to its promising properties in combination with green features, such as renewability and biodegradability, nanocellulose is being intensely studied with regard to a very broad variety of applications, such as packaging [17,18], foams [19,20], water purification [21,22], spun fibers [23], and reinforced polymers [24].

A number of studies showed that addition of nanocellulose to film-forming coatings provides significant improvements in terms of mechanical resistance, whereas, on the downside, a reduction of gloss and increased surface roughness are also reported [25,26,27,28,29,30,31,32,33,34,35]. In addition to changes in final product properties, the processing of coating formulations is also affected by nanocellulose addition in terms of a significant increase in viscosity [35]. This may be of advantage with regard to achieving a stable non-dripping wet-film, but is mostly perceived disadvantageous because it may hinder or obstruct spray-application to surfaces and handling in general.

In the present study, we assess the effect of nanocellulose addition to a linseed oil-based wood coating. In order to provide compatibility with this hydrophobic matrix, nanocellulose is chemically modified with two acid anhydrides of different chain length. Propylene carbonate (PC) was used as the primary solvent for modification of NFC due its good dissolution properties, high boiling point, and low toxicity. PC exhibits a high dipole moment of 16.7 × 10^−30^ cm [36] making it a powerful solvent/swelling agent for polar substances. Based on the hypothesis that modified nanocellulose will enable the formation of a thin protective composite film on the wood surface, significantly improved mechanical resistance of coated wood substrates is expected.

## 2. Materials and Methods

### 2.1. Materials

NFC was purchased from University of Maine (Orono, ME, USA) as an aqueous fibril suspension with a solid content of about 3 wt %. Chemical modification of NFC was performed with acetic anhydride (AA, ≥99% purity, Carl Roth, Karlsruhe, Germany) and (2-dodecen-1-yl)succinic anhydride (DDSA, 95% purity, Sigma-Aldrich, St. Louis, MO, USA) with pyridine (anhydrous, 99.8% purity, Sigma-Aldrich, St. Louis, MO, USA) and 4-(dimethylamino)pyridine (4-DMAP, ≥99% purity, Carl Roth, Karlsruhe, Germany) as catalysts. Solvents used were acetone (≥99.5% purity), propylene carbonate (≥99.7% purity), ethanol (≥99.8% purity, denatured), and *n*-hexane (≥99% purity), all purchased from Carl Roth (Karlsruhe, Germany). For sedimentation tests and wood coating, a commercial linseed oil varnish (Tiger Leinölfirnis, Tiger Coatings GmbH and Co. KG., Wels, Austria) was used along with the thinner Terptin Extra from the same manufacturer. The thinner used is a mixture of solvents, mainly hydrotreated heavy naphtha (petroleum) and 2-methylpropan-1-ol.

### 2.2. Chemical Modification of Nanocellulose

In the first step, NFC was gradually transferred to acetone and propylene carbonate by a stepwise solvent exchange procedure consisting of a filtration step using a Büchner funnel with a Rotilabo Typ 113A filter paper (Carl Roth, Karlsruhe, Germany) and subsequent refilling with fresh solvent (acetone or PC, respectively). After a total of eight filtration steps, an NFC/PC suspension with a solid content of around 4% was received. For chemical modification, approximately 200 g of this suspension (corresponding to 8.0 g of dry cellulose) were weighed into a 1000 mL round bottom flask. Additional PC (200 g) was added and NFC was dispersed homogeneously by continuous stirring with a magnetic stirrer (300 min^−1^). A catalyst solution was prepared by dissolving 0.4 g 4-DMAP in 4.0 g of pyridine and the resulting solution was directly placed in the flask. Thereafter, 40.0 g of the respective anhydride were added to the mixture, corresponding to a 5:1 weight ratio between anhydride and dry cellulose. The flask was heated to 80 °C for a given reaction time (2, 8, and 24 h, respectively) after which the flask was immediately placed in an ice bath to stop the reaction. Modified NFC was repeatedly washed with ethanol to remove unreacted compounds. To evaluate the swelling effect of propylene carbonate on NFC, two different reference samples were prepared with unmodified NFC. While the first reference (AC) was taken immediately after the solvent exchange to acetone, the second reference sample (PC) was further transferred to propylene carbonate and repeatedly washed with ethanol. In order to transfer cellulose fibrils to linseed oil varnish, all samples were further solvent exchanged with *n*-hexane and Terptin Extra thinner.

### 2.3. Characterisation of Modified Nanocellulose

#### 2.3.1. Attenuated Total Reflectance Fourier Transform Infrared Spectroscopy (ATR-FTIR)

For ATR-FTIR measurements, the NFC/hexane suspensions were initially air dried at ambient temperature in a fume hood and stored over silica gel desiccant for three days. Exactly 1.0 g of dry sample was weighed into a flat mould and pressed with a pellet press (Atlas 15T, Specac Ltd., Orpington, UK) using a load of 10 tons for 30 s to form a circular pellet of 13 mm diameter. Three pellets were produced for each of the cellulose variants and placed on the ATR crystal (Zn/Se) of a PerkinElmer Frontier FTIR spectrometer (Perkin Elmer, Waltham, MA, USA). Each pellet was scanned three times from 4000–650 cm^−1^ with a resolution of 4 cm^−1^ after which an average spectrum was calculated. For comparison, all spectra were adjusted to the same baseline and normalized to the C–O stretching vibration of the glucopyranose ring at about 1055 cm^−1^ using the software Spectrum 10™ (PerkinElmer, Waltham, MA, USA).

#### 2.3.2. ^13^C Cross-Polarization Magic Angle Spinning (CP-MAS) NMR Spectroscopy

All solid-state NMR experiments were performed on a Bruker Avance III HD 400 spectrometer (resonance frequency 400.13 MHz for ^1^H, 100.61 MHz for ^13^C, Billerica, MA, USA), equipped with a 4 mm dual broadband cross-polarization magic angle spinning (CP-MAS) probe. ^13^C spectra were acquired with the total sideband suppression (TOSS) sequence at ambient temperature with a spinning rate of 5 kHz, a cross-polarization (CP) contact time of 2 ms, a recycle delay of 2 s, SPINAL-64 ^1^H decoupling and an acquisition time of 43 ms. Chemical shifts were referenced externally against the carbonyl signal of glycine at δ = 176.03 ppm. The acquired free induction decays (FIDs) were apodized with an exponential function (l b = 11 Hz) before the Fourier transformation.

#### 2.3.3. Scanning Electron Microscopy (SEM)

The NFC/hexane suspensions were further diluted with *n*-hexane to a solid content of 0.1 wt % and dispersed by a hand blender (Ultra Turrax T10, IKA, Staufen im Breisgau, Germany) operated at 20,000 min^−1^ for 1 min. A drop of diluted suspension was placed on a specimen holder and left to stand until complete evaporation of *n*-hexane. All samples were directly sputter-coated with a platinum layer of 7 nm thickness in argon as a carrier gas at 5 × 10^−2^ mbar by means of a BAL-TEC MED 020 modular high vacuum coating system (BAL-TEC AG, Balzers, Liechtenstein). SEM imaging was carried out on a FEI Nova NanoSEM 230 (FEI, Hillsboro, OR, USA) operated at an accelerating voltage of 5 kV and a working distance of 5 mm.

#### 2.3.4. Wide Angle X-ray Scattering (WAXS)

In order to evaluate possible effects of the chemical modification on cellulose crystallinity, wide angle X-ray scattering was performed using a Rigaku SmartLab five-axis X-ray diffractometer (Tokyo, Japan). The diffractometer was working with Cu-Kα radiation, a parabolic multilayer mirror in the primary beam plus a secondary graphite monochromator. From the diffractograms obtained, an estimate of crystallinity was performed using the simple peak-height method proposed by Segal et al. [37]. Crystallinity indices were calculated from the (002) reflection peaks of cellulose I.

#### 2.3.5. Sedimentation Test

The stability of NFC/linseed oil suspensions was evaluated in a simple sedimentation test. Modified NFC was dispersed in a 9:1 (*w/w*) mixture of linseed oil varnish and *n*-hexane to yield a final cellulose content of 0.25 wt %. The mixtures were poured into glass vials and photographs were taken after 0 h, 1 h, 1 day, and 14 days.

### 2.4. Preparation and Characterisation of Coated Wood Surfaces

#### 2.4.1. Coating of Wood Substrates

Modified NFC was first transferred from *n*-hexane to the thinner Terptin Extra by a further solvent exchange step. Thereafter, the resulting NFC/thinner suspension was mixed with the linseed oil varnish and further Terptin Extra thinner to yield an oil formulation containing 1 wt % dry NFC and 40 wt % thinner (both relating to the amount of linseed oil varnish). The mixtures were homogenised by 20 passes in a high-pressure homogeniser (APV 1000, SPX Flow Technology, Charlotte, NC, USA) operated at 500 bar. The oil formulations were applied to beech wood substrates (*Fagus sylvatica* L.) by compressed air spraying in a quantity of 55 g∙m^−2^. The finished wood samples were conditioned at 20 °C and 65% relative humidity for three weeks before further processing. For light microscopy, small cubes with a size of approx. 3 × 3 × 3 mm were cut from the surface of coated wood substrates. Smooth cross sections were prepared with an ultramicrotome (Leica Ultracut R, Leica Microsystems, Wetzlar, Germany) and investigated using a Zeiss Axioplan 2 incident light microscope (Carl Zeiss Microimaging GmbH, Jena, Germany).

#### 2.4.2. Surface Roughness and Gloss

Surface roughness of coated wood samples was measured with a contact stylus instrument (Form Talysurf Series 2, Rank Taylor Hobson Ltd., Leicester, UK). Surface profiles were recorded with a diamond stylus (tip radius: 2 µm) over a measuring length of 50 mm. For each coating, nine measurements were carried out in the grain direction of the wood. From the surface profile, the average roughness (*R*_a_) was calculated by means of the software Taylor Hobson µltra (Leicester, UK) using a Gaussian filter with a cut-off wavelength of 2.5 mm and a bandwidth ratio of 300:1. Surface gloss was evaluated at an 85° incidence angle using a gloss meter (micro-TRI-gloss, BYK-Gardner GmbH, Geretsried, Germany), again with nine measurements per variant in the grain direction.

#### 2.4.3. Abrasion Testing

For each oil variant, three specimens with a size of 100 mm × 100 mm were cut from the finished wood panels. The surface resistance to mechanical wear was assessed by a newly developed method combining abrasive wear with subsequent water contact angle measurement in the abraded area. Since the oil is gradually removed from the wood surface by abrasion, the contact angle changes from an initially-high value of the pure oil on the surface to lower values characteristic of the subjacent wood with increasing abrasive load. Thus, the decrease in contact angle may be considered as a measure for the surface’s wear resistance. The test specimens were attached to a rotary platform abrasion tester (Taber^®^ Abraser Model 5135, Taber Industries, NY, USA) equipped with S42 sandpaper and subjected to abrasive wear as described in the European standard ÖNORM EN 438-2 [38]. In total, 150 abrasion cycles (rotations) on the Taber Abraser were performed with contact angle measurements after 0, 5, 10, 15, 20, 25, 50, 75, 100, 125, and 150 cycles.

## 3. Results and Discussion

**Figure 1:**
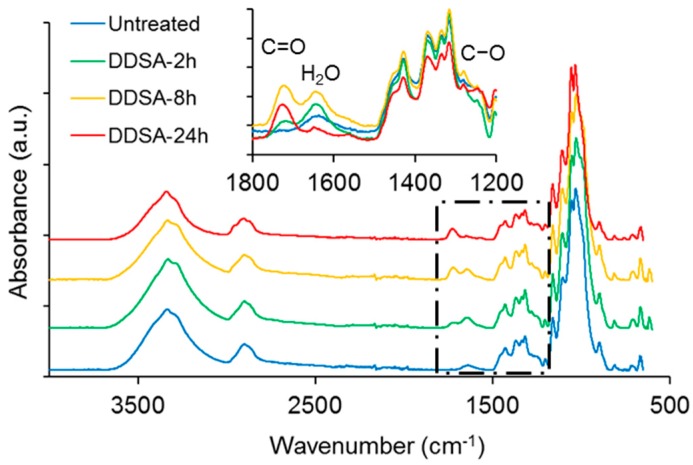

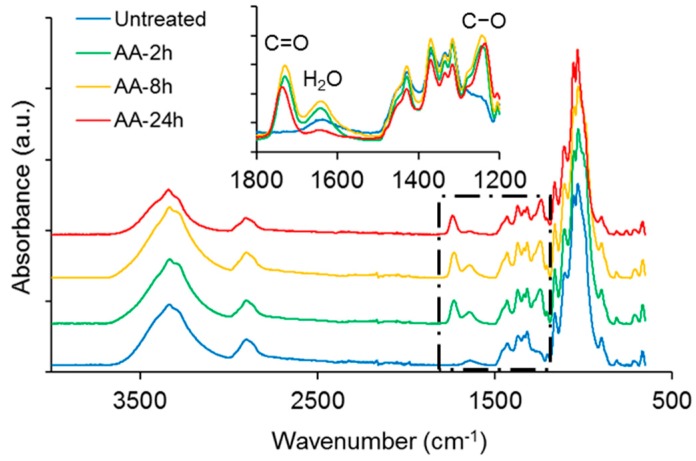
Attenuated Total Reflectance-Fourier Transform Infrared (ATR-FTIR) spectra of nanofibrillated cellulose (NFC) modified with (2-dodecen-1-yl)succinic anhydride (DDSA) and acetic anhydride (AA), respectively, compared to unmodified NFC.

ATR-FTIR spectra of NFC modified with acetic or succinic anhydride, respectively, are shown in comparison to untreated NFC in Figure 1. Overall, the spectra look very similar and major changes indicating hydrophobisation, e.g., a significant reduction in absorbance of the broad –OH band at approx. 3340 cm^−1^, are not evident, with the exception of the variants treated for 24 h. However, a closer inspection of the spectra in the region between 1800 cm^−1^ and 1200 cm^−1^ reveals further differences between the individual modification variants. Both, DDSA- and AA-modified samples show more or less pronounced absorption peaks at around 1730 cm^−1^ and 1640 cm^−1^, attributable to C=O stretching [39] and water associated with cellulose [40], respectively. A clear difference between DDSA and AA-modified samples can be seen at 1240 cm^−1^ where the latter show a distinct band associated to carbonyl C–O [41] which is missing for DDSA-modified samples. For assessing the degree of modification, a simple peak height ratio was calculated by relating the peak height of the carbonyl band at 1730 cm^−1^ to the C–O stretching in cellulose at 1055 cm^−1^ (Table 1). Here, a clear relative increase is to be observed for the modified variants as related to unmodified NFC, indicating that all samples have been successfully modified, albeit to rather low extent. The low degree of modification becomes even more obvious when the peak height ratios found in this study are compared to literature data, where peak absorption ratios (*I*_1740_/*I*_1060_) of up to 0.5 were reported for acetylated cellulose nanocrystals [42,43]. When the two modification variants used in the present study are compared to each other, it appears that AA-modification proceeds faster and to a higher extent than modification with DDSA.

**Figure 2:**
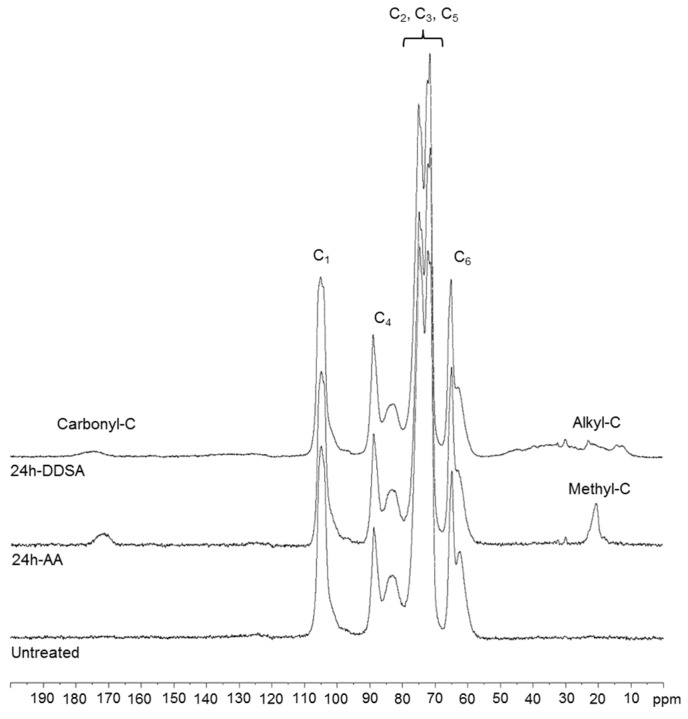
^13^C Cross-Polarization Magic Angle Spinning Nuclear Magnetic Resonance (CP-MAS NMR) spectra of unmodified NFC compared to NFC modified with DDSA and AA, respectively, for 24 h.

^13^C solid-state NMR spectra (Figure 2) provide clear evidence of the chemical modification for both the alkylmaleic anhydride and the acetic anhydride modification. Besides the resonances of cellulose carbons (C_1_–C_6_) between 63 and 105 ppm, resonances of the alkyl chain substituents in DDSA-modified samples are clearly visible in the region of 20–40 ppm, while the acetyl´s methyl group resonates at 20.3 ppm. The ester carbonyls appear in both samples at around 170–174 ppm, as expected. These results are fully in line with the outcome of the IR measurements.

**Figure 3:**
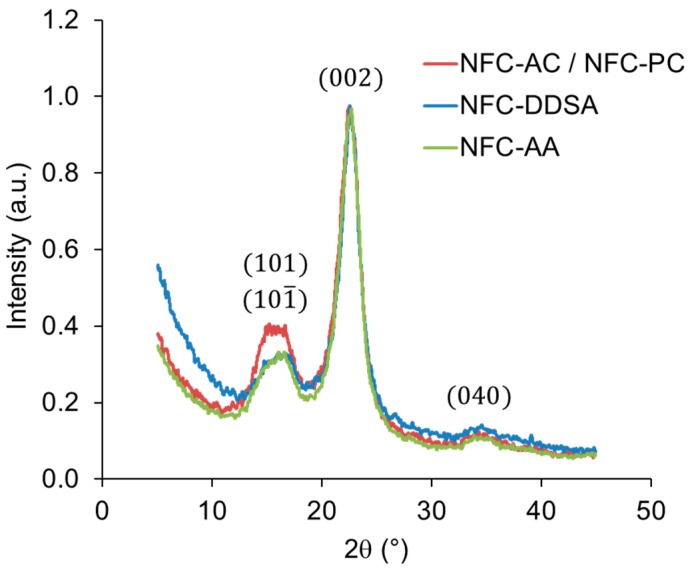
X-ray diffractograms of unmodified NFC (NFC-PC/NFC) compared to NFC modified with acetic anhydride (NFC-AA) and (2-dodecen-1-yl)succinic anhydride (NFC-DDSA) for 24 h.

In order to study the potential effects of the chemical treatment on the structure of NFC, WAXS measurements were performed. The diffraction patterns of untreated NFC and NFC solvent-exchanged to propylene carbonate are virtually identical, indicating no XRD-relevant structural change (Figure 3). Contrary to that, both chemically-modified variants with 24 h reaction time show minor changes in their diffraction patterns compared to the reference material. Thus, some small differences were also found in crystallinity indices, being 0.76, 0.73, and 0.80 for unmodified NFC, NFC-DDSA, and NFC-AA, respectively. While the most intense peak associated with the cellulose I (002) crystalline plane seems unaffected, the intensity of the overlapping cellulose (101)/($10\bar{1}$) peak is clearly reduced in both modified specimens. In simple terms, this may be interpreted as a thinning of crystals along the direction perpendicular to this diffraction plane resulting from some degradation of NFC during the modification treatment.

**Figure 4:**
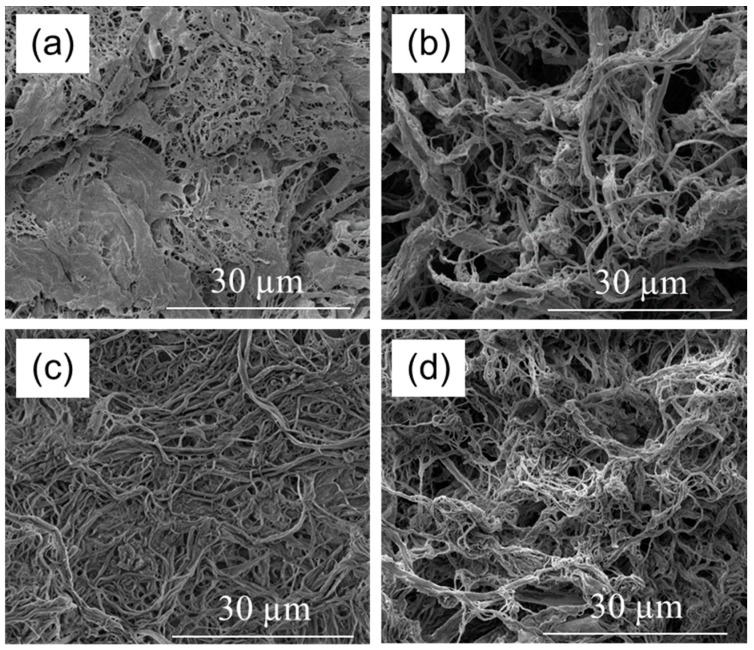
Scanning electron microscopy (SEM) images of different NFC variants dried from *n*-hexane: (**a**) unmodified NFC; (**b**) blank NFC sample, solvent-exchanged to propylene carbonate; (**c**) 24 h (2-dodecen-1-yl)succinic anhydride-treated NFC; and (**d**) 24 h acetic anhydride-treated NFC.

The indication of a certain degree of surface-chemical modification towards reduced surface-chemical polarity provided by FTIR and NMR results is supported by differences observed in the morphology of NFC variants after drying from *n*-hexane, as revealed by SEM (Figure 4). When comparing the two modified variants (Figure 4c,d) with the unmodified reference (Figure 4a), a more fibrillary and less compact structure of the modified variants is clearly visible. This may be interpreted as the result of reduced agglomeration of NFC during drying due to a reduced number of free OH groups after chemical modification. Interestingly, the blank NFC sample (Figure 4b), which was transferred to propylene carbonate, but not subjected to chemical modification, also shows a clearly more fibrillary character than the untreated reference.

**Figure 5:**
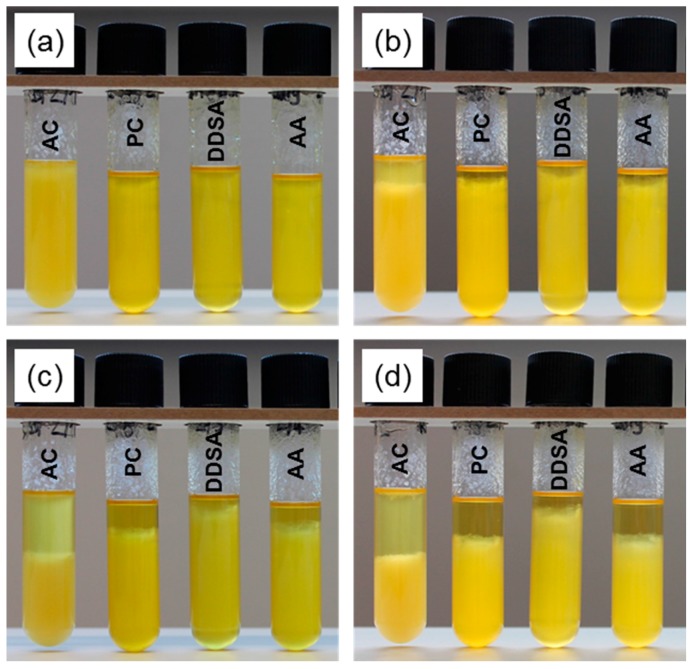
Stability of NFC/linseed oil suspensions after 0 h (**a**); 1 h (**b**); 1 day (**c**); and 14 days (**d**) sedimentation time (AC: NFC solvent exchanged to acetone, PC: NFC solvent exchanged to propylene carbonate, DDSA: NFC modified with (2-dodecen-1-yl)succinic anhydride for 24 h, AA: NFC modified with acetic anhydride for 24 h).

Complementary to SEM, a sedimentation trial in linseed oil was also performed with the same variants (Figure 5) in order to study the stability of suspensions of these NFC samples, which may serve as an indication of surface-chemical compatibility between linseed oil and the respective NFC variant. In an ascending order of stability, AC performs the least stable, PC and AA perform at an intermediate level, and DDSA clearly performs the best, i.e., it shows the lowest sedimentation in linseed oil. The good stability of the latter variant is not consistent with FTIR results, where clearer indications of successful modification were found for AA compared to DDSA. However, contrary to the modification with acetic anhydride, a bulky and highly hydrophobic side chain is transferred to cellulose in DDSA modification. Due to the bulkiness of the DDSA molecule, the reaction with cellulose is expected to occur to a smaller extent than for AA, although its hydrophobisation effect may be significantly higher. A higher degree of hydrophobicity would cause better surface-chemical compatibility between modified NFC and linseed oil, explaining the better performance of DDSA-NFC in sedimentation testing. Due to its loose structure (Figure 4b), unmodified NFC, which was also suspended in PC, performed clearly better than unmodified NFC suspended in acetone. It is assumed that residual PC, which is tightly surface-bound by strong hydrogen bonds and has replaced some water molecules at the cellulose surface, hinders structural collapse (“hornification”) that otherwise readily occurs upon drying of non-solvent-exchanged NFC. Highly fibrillar structures are, thus, better preserved upon drying.

**Figure 6:**
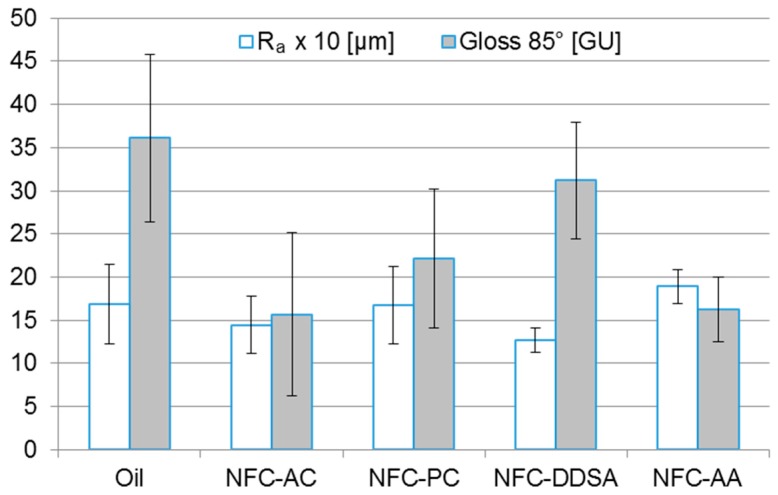
Arithmetic mean roughness (*R*_a_) and 85° gloss level of beech wood surfaces coated with linseed oil varnish containing 1 wt % NFC.

Wood surfaces coated with different linseed oil formulations were characterised with regard to surface gloss and roughness as well as resistance to mechanical abrasion. As obvious from Figure 6, the addition of 1 wt % NFC had no significant effect on the average surface roughness of oiled wood surfaces. All NFC-modified coatings showed a surface roughness similar to the unmodified oil coating, which is presumably due to the comparably high surface roughness of the subjacent wood substrate. By contrast, considerable differences in surface gloss were found for the individual variants. With the exception of NFC-DDSA, all cellulose-modified coatings appeared clearly less glossy than the unmodified oil. This matting effect of NFC has already been described for film-forming wood coatings [28,30] and was primarily attributed to an increased surface roughness induced by the addition of NFC. Surface gloss is usually determined by the refractive index of the material, the incident angle of light, and the surface topography [44]. Since the incident angle was the same for all measurements and surface roughness was similar for all variants, the higher gloss level of NFC-DDSA may be explained by a change in the refractive index of NFC introduced by DDSA modification. However, this was not investigated in more detail.

**Figure 7:**
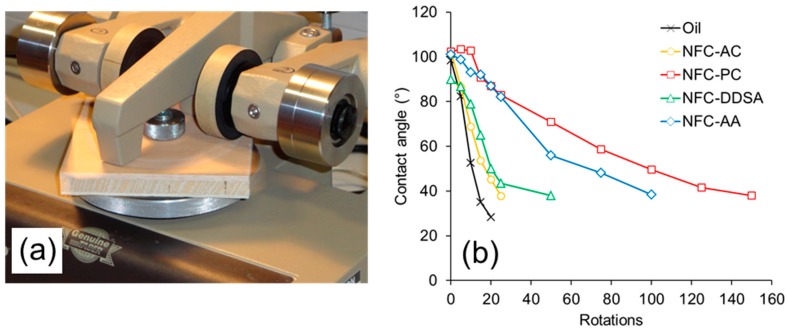
Wear resistance of oiled wood surfaces containing 1 wt % NFC. The surfaces were subjected to abrasive wear on a Taber^®^ Abraser (**a**) and subsequent water contact angle measurements in the abraded area. A higher contact angle after a given number of abrasion cycles indicates a higher wear resistance (**b**).

The wear resistance of coated wood surfaces was evaluated by a test combining abrasive loading on a Taber Abraser and subsequent wettability measurements (Figure 7). Before the first abrasion cycle, all specimens show similar wettability with water as expressed by a contact angle of approximately 100°. With increasing number of abrasion cycles, i.e., rotations of the Abraser tool on the surface, all specimens show a loss of hydrophobisation due to the gradual removal of oil and oil-impregnated wood cells, respectively, from the surface. This loss of water repellence is most pronounced and quick for the samples coated with unmodified linseed oil. As compared to the blank oil, formulations containing NFC-AC and NFC-DDSA show a slight improvement, whereas the AA-modified variant shows a significantly prolonged preservation of water repellence after abrasion treatment. Surprisingly, linseed oil containing unmodified NFC that was transferred to propylene carbonate prior to incorporation into linseed oil clearly performs best among the variants studied. Apparently, the abrasion resistance of cured linseed oil containing modified NFC does not directly relate to the stability of liquid oil formulations, but there seems to be a correlation to the network structure of NFC present after drying from non-polar media as evident from SEM images. As can be seen from Figure 4, the density of the fibril network differs significantly among the individual NFC types and decreases in the order NFC-AC, NFC-DDSA, NFC-AA, and NFC-PC which corresponds well to the results of abrasion testing. Thus, a loose fibril network as apparent for NFC-PC seems to be beneficial to wear resistance, most likely due to a more homogeneous distribution of cellulose fibrils in linseed oil even after drying.

**Figure 8:**
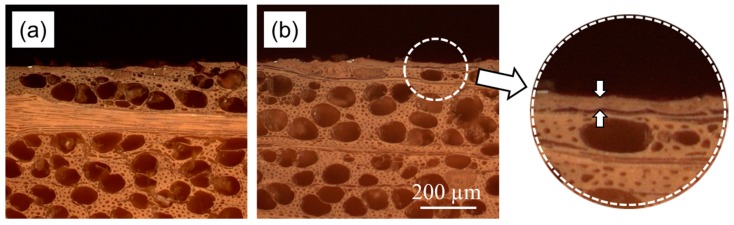
Representative light-microscopic images of cross sections of beech wood treated with (**a**) linseed oil varnish and (**b**) linseed oil varnish modified with NFC solvent-exchanged to propylene carbonate. The inset highlights the oil film obtained using this modified oil formulation.

To prove the hypothesis that NFC-modified linseed oil forms a protective layer on the wood surface, light microscopy was performed (Figure 8). When a wood surface is treated with oil-based coatings, usually no distinct coating film is formed (Figure 8a). Instead, the oil penetrates into the wood, thus providing it with limited water repellence and a pleasant appearance. In the case of NFC-modified oil, a thin film of a few tens of microns is formed on top of the treated surface (Figure 8b). We propose that this film is formed because NFC prevents full penetration of the applied oil into the bulk of the wood, resulting in a protective composite layer of NFC-reinforced dried oil. The formation of such a protective layer was observed with all NFC-modified variants, but it was most pronounced in the case of NFC-PC. It is assumed that this layer maintains water repellence to a much higher extent than in the case of unmodified oil-coated surfaces lacking such a protective layer (Figure 7).

## 4. Conclusions

As revealed by FTIR and NMR measurements, NFC was successfully modified with acetic anhydride and (2-dodecen-1-yl)succinic anhydride, respectively, using propylene carbonate as a solvent. When mixed with linseed oil varnish, suspensions containing DDSA-modified NFC showed the highest stability in sedimentation tests, indicating good surface compatibilization of cellulose. However, in abrasion tests carried out on coated wood surfaces, acetylated NFC and, surprisingly, unmodified NFC suspended in propylene carbonate, clearly performed better. Contrary to acetone, highly-polar propylene carbonate seems to more effectively preserve the fibrillary structure of NFC upon drying from non-polar media. Furthermore, NFC was shown to retain oil on the wood surface, resulting in the formation of a thin NFC/oil composite layer on the surface which acts as a more or less effective protection against water and mechanical influences.

## Figures and Tables

**Table 1:** Peak height ratio (*I*_1730_/*I*_1055_) of DDSA- and AA-modified NFC samples as derived from ATR-FTIR measurements as a function of reaction time.

	Untreated	2 h	8 h	24 h
DDSA	0.04	0.06	0.12	0.09
AA	0.04	0.16	0.18	0.13

## References

[1] Pizzi A. (1989). Research vs. Industrial Practice with Tannin-Based Adhesives. Adhesives from Renewable Resources.

[2] Ferdosian F., Pan Z., Gao G., Zhao B. (2017). Bio-based adhesives and evaluation for wood composites application. Polymers.

[3] Philipp C. (2010). The future of wood coatings. Eur. Coat. J..

[4] Challener C. (2015). Trends in interior wood coatings: Tracking the shift from solvent to waterborne and uv. JCT Coat. Technol..

[5] Alam M., Akram D., Sharmin E., Zafar F., Ahmad S. (2014). Vegetable oil based eco-friendly coating materials: A review article. Arab. J. Chem..

[6] Lazzari M., Chiantore O. (1999). Drying and oxidative degradation of linseed oil. Polym. Degrad. Stab..

[7] Nikolic M., Lawther J.M., Sanadi A.R. (2015). Use of nanofillers in wood coatings: A scientific review. J. Coat. Technol. Res..

[8] Pilotek S., Tabellion F. (2005). Nanoparticles in coatings. Eur. Coat. J..

[9] Nejad M., Cooper P., Landry V., Blanchet P., Koubaa A. (2015). Studying dispersion quality of nanoparticles into a bio-based coating. Prog. Org. Coat..

[10] Dufresne A. (2013). Nanocellulose: A new ageless bionanomaterial. Mater. Today.

[11] Saito T., Kuramae R., Wohlert J., Berglund L.A., Isogai A. (2013). An ultrastrong nanofibrillar biomaterial: The strength of single cellulose nanofibrils revealed via sonication-induced fragmentation. Biomacromolecules.

[12] Oksman K., Aitomäki Y., Mathew A.P., Siqueira G., Zhou Q., Butylina S., Tanpichai S., Zhou X., Hooshmand S. (2016). Review of the recent developments in cellulose nanocomposite processing. Compos. A Appl. Sci. Manuf..

[13] Habibi Y. (2014). Key advances in the chemical modification of nanocelluloses. Chem. Soc. Rev..

[14] Lindström T. (2017). Aspects on nanofibrillated cellulose (nfc) processing, rheology and nfc-film properties. Curr. Opin. Colloid Interface Sci..

[15] Li M.C., Wu Q., Song K., Lee S., Qing Y., Wu Y. (2015). Cellulose nanoparticles: Structure-morphology-rheology relationships. ACS Sustain. Chem. Eng..

[16] Xu Y., Atrens A.D., Stokes J.R. (2017). Rheology and microstructure of aqueous suspensions of nanocrystalline cellulose rods. J. Colloid Interface Sci..

[17] Ferrer A., Pal L., Hubbe M. (2017). Nanocellulose in packaging: Advances in barrier layer technologies. Ind. Crops Prod..

[18] Li F., Mascheroni E., Piergiovanni L. (2015). The potential of nanocellulose in the packaging field: A review. Packag. Technol. Sci..

[19] Cervin N.T., Andersson L., Ng J.B.S., Olin P., Bergström L., Waìšgberg L. (2013). Lightweight and strong cellulose materials made from aqueous foams stabilized by nanofibrillated cellulose. Biomacromolecules.

[20] Blaker J.J., Lee K.Y., Li X., Menner A., Bismarck A. (2009). Renewable nanocomposite polymer foams synthesized from pickering emulsion templates. Green Chem..

[21] Carpenter A.W., De Lannoy C.F., Wiesner M.R. (2015). Cellulose nanomaterials in water treatment technologies. Environ. Sci. Technol..

[22] Voisin H., Bergström L., Liu P., Mathew A.P. (2017). Nanocellulose-based materials for water purification. Nanomaterials.

[23] Clemons C. (2016). Nanocellulose in spun continuous fibers: A review and future outlook. J. Renew. Mater..

[24] Lee K.Y., Aitomäki Y., Berglund L.A., Oksman K., Bismarck A. (2014). On the use of nanocellulose as reinforcement in polymer matrix composites. Compos. Sci. Technol..

[25] Vardanyan V., Galstian T., Riedl B. (2015). Characterization of cellulose nanocrystals dispersion in varnishes by backscattering of laser light. J. Coat. Technol. Res..

[26] Landry V., Blanchet P. (2011). Coatings Containing Nanocrystalline Cellulose, Processes for Preparation and Use Thereof. Patent.

[27] Vardanyan V., Galstian T., Riedl B. (2015). Effect of addition of cellulose nanocrystals to wood coatings on color changes and surface roughness due to accelerated weathering. J. Coat. Technol. Res..

[28] Veigel S., Grüll G., Pinkl S., Obersriebnig M., Müller U., Gindl-Altmutter W. (2014). Improving the mechanical resistance of waterborne wood coatings by adding cellulose nanofibres. React. Funct. Polym..

[29] Vardanyan V., Poaty B., Chauve G., Landry V., Galstian T., Riedl B. (2014). Mechanical properties of uv-waterborne varnishes reinforced by cellulose nanocrystals. J. Coat. Technol. Res..

[30] Poaty B., Vardanyan V., Wilczak L., Chauve G., Riedl B. (2014). Modification of cellulose nanocrystals as reinforcement derivatives for wood coatings. Prog. Org. Coat..

[31] Vlad-Cristea M.S., Landry V., Blanchet P., Ouellet-Plamondon C. (2013). Nanocrystalline cellulose as effect pigment in clear coatings for wood. ISRN Nanomater..

[32] Grüneberger F., Künniger T., Huch A., Zimmermann T., Arnold M. (2015). Nanofibrillated cellulose in wood coatings: Dispersion and stabilization of zno as uv absorber. Prog. Org. Coat..

[33] Grüneberger F., Künniger T., Zimmermann T., Arnold M. (2014). Nanofibrillated cellulose in wood coatings: Mechanical properties of free composite films. J. Mater. Sci..

[34] Kaboorani A., Auclair N., Riedl B., Landry V. (2016). Physical and morphological properties of uv-cured cellulose nanocrystal (cnc) based nanocomposite coatings for wood furniture. Prog. Org. Coat..

[35] Grüneberger F., Künniger T., Zimmermann T., Arnold M. (2014). Rheology of nanofibrillated cellulose/acrylate systems for coating applications. Cellulose.

[36] Reichardt C., Welton T. (2010). Solvents and Solvent Effects in Organic Chemistry.

[37] Segal L., Creely J.J., Martin A.E., Conrad C.M. (1959). An empirical method for estimating the degree of crystallinity of native cellulose using the x-ray diffractometer. Text. Res. J..

[38] (2016). Önorm EN 438-2 High-Pressure Decorative Laminates (HPL)—Sheets Based on Thermosetting Resins (Usually Called Laminates)—Part 2: Determination of Properties.

[39] Popescu C.M., Popescu M.C., Singurel G., Vasile C., Argyropoulos D.S., Willfor S. (2007). Spectral characterization of eucalyptus wood. Appl. Spectrosc..

[40] Fortier C., Montalvo J., Hoven T.V., Easson M., Rodgers J., Condon B. (2014). Preliminary evidence of oxidation in standard oven drying of cotton: Attenuated total reflectance/fourier transform infrared spectroscopy, colorimetry, and particulate matter formation. Text. Res. J..

[41] Fan Q.G., Lewis D.M., Tapley K.N. (2001). Characterization of cellulose aldehyde using fourier transform infrared spectroscopy. J. Appl. Polym. Sci..

[42] Çetin N.S., Tingaut P., Özmen N., Henry N., Harper D., Dadmun M., Sèbe G. (2009). Acetylation of cellulose nanowhiskers with vinyl acetate under moderate conditions. Macromol. Biosci..

[43] Yan M., Li S., Zhang M., Li C., Dong F., Li W. (2013). Characterization of surface acetylated nanocrystalline cellulose by single-step method. BioResources.

[44] Bulian F., Graystone J.A. (2009). Wood Coatings: Theory and Practice.

